# Interhemispheric transcallosal transchoroidal approach to a pineal teratoma in a 15-year-old boy

**DOI:** 10.3171/2021.4.FOCVID2126

**Published:** 2021-07-01

**Authors:** Giuseppe Cinalli, Maria Rosaria Scala, Alessandra Marini, Alessia Imperato, Giuseppe Mirone, Pietro Spennato

**Affiliations:** 1 Department of Pediatric Neurosurgery, Santobono-Pausilipon Children’s Hospital, Naples; and; 2 Division of Neurosurgery, Department of Neurosciences, Reproductive and Odonotostomatological Sciences, Università degli Studi di Napoli “Federico II,” Naples, Italy

**Keywords:** teratoma, child, third ventricle, interhemispheric transcallosal transchoroidal approach, Herophilus-Galen line, neuroendoscopy

## Abstract

In this video, the authors present an interhemispheric transcallosal transchoroidal approach to a pineal mass in a 15-year-old boy. He received emergency endoscopic third ventriculostomy (ETV), then an endoscopic biopsy that revealed an immature teratoma. Surgical removal was selected. The mass was located very high in the posterior third ventricle, hidden behind the splenium of the corpus callosum and the vein of Galen, so an interhemispheric transcallosal approach followed by a complete dissection of the whole choroidal fissure was chosen and allowed complete removal of the tumor. Microsurgical dissection is presented, showing clearly in detail all the neurovascular structures encountered.

The video can be found here: https://stream.cadmore.media/r10.3171/2021.4.FOCVID2126.

## Transcript

### 0:20 Clinical Presentation

In this video, we present an interhemispheric transcallosal transchoroidal approach to a pineal teratoma in a 15-year-old boy. The patient presented a 2-week history of headache. He was referred from another hospital after emergency ETV was performed for acute triventricular hydrocephalus due to a pineal mass. Neurological exam was normal.

### 0:40 Radiology and Ancillary Tests

Craniospinal MRI showed resolution of the triventricular hydrocephalus and the presence of an ETV track. The mass was evident in the pineal region and posterior third ventricle without CSF seeding. Blood and CSF alpha-fetoprotein, beta-hCG, and placental alkaline phosphatase were within normal limits.

### 1:00 Biopsy

Endoscopic biopsy was performed through a more anterior burr hole passing below the massa intermedia revealing immature teratoma. On the base of histology, surgical removal was decided. To select the approach, sagittal midline MRI image was chosen.

### 1:15 Surgical Strategy Exclusion of OITA Approach

The mass was located very high in the posterior third ventricle hidden behind the splenium and the vein of Galen. The lowest point of the vein of Galen was identified; then the highest point of the torcular Herophili was identified. The line joining these two points, the Herophilus-Galen line, identifies the highest possible line of sight of a microscope when performing an occipital interhemispheric transtentorial (OITA) approach. In this case, the OITA approach would allow excellent control of the lower half of the tumor, but the upper half would remain hidden behind the splenium and the vein of Galen. 3D model showing the Herophilus-Galen line helps to understand how deep and hidden is the tumor behind the Galen complex without improvements of vision moving the microscope.

### 2:00 Surgical Strategy Decision for Transchoroidal Approach

This lesion was highly located in the posterior third ventricle, so an interhemispheric transcallosal approach followed by a complete dissection of the whole choroidal fissure would allow complete exposure of the tumor mass to the line of sight of the operating microscope.

3D model helps to understand that a transcallosal approach with choroidal fissure dissection from above working between the two internal cerebral veins would allow complete control of the tumor from the anterior to the posterior pole. The more posterior trajectory seems to cross the posterior column of the fornix, but dissection of the choroid fissure and the section of the anterior septal vein allows a very lateral displacement of the right internal cerebral vein, creating a very large operative corridor that allows to work always laterally to the posterior column of the fornix, minimizing the risks to injure it.

### 2:46 Position and Flap

Position is supine, with mild flexion of the head. The standard transcallosal approach is performed through a flap that is one-third behind the coronal two-thirds anterior to the coronal crossing the midline, allowing perfect visualization of the tumor.

### 3:01 Interhemispheric Dissection

Interhemispheric approach allows visualization of the two cingulate gyri and of the two calloso-marginal arteries. The two cingulate gyri can be very adherent, and this requires a very slow and careful dissection in order to avoid any injury to them, but with smooth and careful dissection, it is possible to expose in the depth the corpus callosum that should be dissected for a length of at least 3 cm.^1,2^ Here we see the corpus callosum, the left calloso-marginal arteries, and the two cingulate gyri.

### 3:36 Callosotomy and Vein Identification

The self-retaining retractor is repositioned deeper in order to expose correctly the corpus callosum that is separated with bipolar coagulation until opening of the ependyma of the right lateral ventricle showing the choroid plexus in the depth. After completion of the 2-cm callosotomy, the choroid plexus is evident with the right thalamostriate vein, the fornix, and the right thalamus.^3^ Choroid fissure is hidden below the choroid plexus that needs to be coagulated from the foramen of Monro to the posterior aspect of the thalamus in order to unveil the choroidal fissure that is below, dissecting the connections between the two internal cerebral veins, the thalamostriate vein, and the anterior septal vein.

### 4:17 Vein Dissection

These veins must be dissected carefully, but in a very clear way in order to coagulate the anterior septal vein. Here you see that we are dissecting the internal cerebral vein from the fornix, discovering the choroid fissure below. Here you can see the taenia fornicis, the right internal cerebral vein, the anterior septal vein, and the right thalamus, and further dissection is necessary for complete identification.^4,5^

### 4:57 Sacrifice of Anterior Septal Vein

At this point, the junction between the right thalamostriate vein, the anterior septal vein, and the internal cerebral vein is more clear. Complete dissection of the anterior septal vein is necessary before coagulation. The sacrifice of this vein allows complete opening of the choroid fissure. Here you see that we spend a lot of time to separate this vein from the ependyma in order to avoid thermal injury to the underlying fornix at the moment of coagulation. This is the moment of the section of the anterior septal vein that finally allows the complete opening of the anterior part of the choroid fissure that at this moment is still covered by choroid plexus.

### 5:41 Dissection of Choroid Fissure

We coagulate slowly the choroid plexus at the level of the foramen of Monro in order to have complete vision of the anterior part of the third ventricle. You can see the tumor below, and repositioning of the self-retaining retractor allows a nice view of the more anterior part of the tumor. At this point, the dissection continues by coagulating and separating the small vessels that run between the two internal cerebral veins at the level of the tela choroidea of the third ventricle. You can clearly see the very thin layer of the tela choroidea, with very small vessels that must be coagulated and the relationship of the internal cerebral vein, the tela choroidea, and tumor underlying the tela choroidea.

### 6:41 Tumor Biopsy

This dissection is the most careful part of the whole surgical procedure. You can see very nicely the thin layer of the tela choroidea that needs to be opened progressively with very thin vessels that need to be coagulated and separated before having access to the capsule of the tumor. This process is continued throughout the length of the choroidal fissure. The white capsule of the tumor is very evident below the last remnants of the tela choroidea, and at this time we coagulate, we open the capsule and do a biopsy of the inner part of the tumor.

### 7:22 Tumor Debulking and Further Dissection

Then we start the internal decompression with the ultrasonic surgical aspirator. It is clear the relationship with the massa intermedia, and at this point we dissect the anterior pole of the tumor from below the massa intermedia. The adhesions are relatively loose, and the tumor can be easily dissected from the lower aspect of the massa intermedia until identification of the more anterior part of the third ventricle. Then dissection continues on the right side until we reach the posterior pole of the tumor.

### 8:02 Lateral and Posterior Dissection

Here the posterior pole is dissected from below the more posterior part of the tela choroidea. This is the relationship with the right internal cerebral vein, and the last remnant of tela choroidea are opened with scissors in order to expose the posterior pole of the tumor. Then we go laterally on the left side, dissecting the left lateral aspect of the tumor, and then we go back on the right side in order to finally find the cavity of the third ventricle, and because of the significant reduction of the mass due to the internal debulking, we are finally able, following dissection of the posterior and lateral edges of the tumor, to dissect the tumor from the lateral wall of the third ventricle and find the cavity of the third ventricle.

### 8:56 Tumor Removal

Then we continue the dissection anteriorly. We find the floor of the third ventricle, and we dissect the tumor from the floor of the third ventricle until obtaining a complete freedom of the residual tumor inside the cavity that can be fully and completely removed in block with the forceps. This is the immediate postoperative MRI showing the approach and the complete removal of the tumor.

### 9:23 Histology and Outcome

The histology showed immature teratoma with areas of germinoma. Adjuvant treatment was chemotherapy plus radiotherapy according with SIOP CNS GCT II protocol. Clinical outcome was a normal neurological exam with no evidence of disease 24 months after surgery as shown by the follow-up MRI at 2 years.

### 9:48 Conclusions

In conclusion, pineal tumors predominantly invading the posterior third ventricle can be difficult to approach through the supracerebellar or interhemispheric transtentorial way. The Herophilus-Galen line of sight is helpful in choosing surgical approach. The interhemispheric transcallosal transchoroidal approach offers complete visual control of the third ventricle from anterior commissure to the quadrigeminal cistern and is electively indicated for tumors of the posterior third ventricle.

## Author Contributions

Primary surgeon: Cinalli. Assistant surgeon: Spennato. Editing and drafting the video and abstract: Cinalli, Scala, Marini, Imperato, Mirone. Critically revising the work: Cinalli, Marini, Imperato, Spennato. Reviewed submitted version of the work: Cinalli, Spennato. Approved the final version of the work on behalf of all authors: Cinalli. Supervision: Cinalli.
